# 

**Published:** 1915-06-19

**Authors:** 


### CONTENTS.

PAGE

**The Medical Profession and the Notification of Births Act** ... 241

**General Medical Council and the Supply of Doctors** ... 242

**Hospital and Institutional News** ... 243
The King and Hospital Sunday—Precautions against Poisonous Gases: A Parisian Formula—The "M.A.B." and Wounded Soldiers—The Maudsley War Hospital—A German Matron: Appeal to the Secretary of State—The Delay in Naturalisation—The Second Military Hospital at Cardiff Opened—Clergymen as Army Drivers—The War and the Epileptic Colony—New Dietaries in Poor-Law Institutions—A New Woman's Post in the Public Health Department—The Lack of Mental Hospital Beds—West End Hospital and the Wounded—Five Hundred Cases for Ten Guineas—The New Sanatoria for London Consumptives—York Hospital and its Finances—Cottage Homes for Poor-Law Children—Anomalies in the Poor-Law Service—Another Rebuff for Recognition of Services—The Medical Staff at Edmonton Military Hospital—Verses, and a Piano—A Curious Offer—The British Pharmaceutical Conference—The Secretaryship of the Hampstead General Hospital—Duty-Free Spirit for Hospitals: Text of the Government Proposals—This Week's Drug Market—To Our Readers.

**Conservancy Methods on Active Service** ... 249
Medical Administration in the Trenches.

**A Father of Modern Hospitals** ... 251
John Billings' "Life"; By Dr. F. H. Garrison.

**The Educative Work of the School Doctor** ... 253
Practical Points on Lectures to Parents.

**Hospital Unification in Cape Town** ... 254

The Method of Control.

**National Insurance Act Developments** ... 255
The Cost to the State of National Health Insurance.

**Round the World** ... 256

Fellow-Workers and the Roll of Honour.

**The Evolution of the Modern Hospital** ... 257

VIII.—The Problem of the Unpaid Staff: By B. Burford Rawlings.

**The Rule in Bathing** ... 258

**The Editor's Letter-Box** ... 259
War Service Badges for Hospital Staffs. Fires due to Air Raids.

**War Service Badges for Hospital Staffs** ... 260
What to Avoid and What To Do.

**The Civil and Military Uses of British Health Resorts** ... iv

**The Institutional Worker** ... (Suppl.) 1

